# Fine particulate matter (PM2.5) inhibits ciliogenesis by increasing SPRR3 expression via c-Jun activation in RPE cells and skin keratinocytes

**DOI:** 10.1038/s41598-019-40670-y

**Published:** 2019-03-08

**Authors:** Ji-Eun Bae, Hyunjung Choi, Dong Woon Shin, Hye-Won Na, Na Yeon Park, Joon Bum Kim, Doo Sin Jo, Min Ji Cho, Jung Ho Lyu, Jeong Ho Chang, Eunjoo H. Lee, Tae Ryong Lee, Hyoung-June Kim, Dong-Hyung Cho

**Affiliations:** 1 School of Life Sciences, Kyungpook National University, Daegu, 41566 Republic of Korea; 2 Graduate School of East-West Medical Science, Kyung Hee University, Yongin, Gyeonggi-do 17104 Republic of Korea; 3 R&D Unit, AmorePacific Corporation, Yongin, Gyeonggi-do 17074 Republic of Korea; 4 Department of Genetic Engineering, Kyung Hee University, Yongin, Gyeonggi-do 17104 Republic of Korea; 5 Department of Biology Education, Kyungpook National University, Daegu, 41566 South Korea

## Abstract

Exposure to fine particulate matter (PM) with diameter <2.5 µm (PM2.5) causes epithelium injury and endothelial dysfunction. Primary cilia are sensory organelles that transmit extracellular signals into intracellular biochemical responses and have roles in physiology. To date, there have been no studies investigating whether PM2.5 affects primary cilia in skin. We addressed this in the present study using normal human epidermal keratinocytes (NHEKs) and retinal pigment epithelium (RPE) cells. We found that formation of primary cilium is increased in differentiated NHEKs. However, treatment with PM2.5 blocked increased ciliogenesis in NHEKs and RPE cells. Furthermore, PM2.5 transcriptionally upregulated small proline rich protein 3 (SPRR3) expression by activating c-Jun, and ectopic expression of SPRR3 inhibits suppressed the ciliogenesis. Accordingly, treatment with c-Jun activator (anisomycin) induced SPRR3 expression, whereas the inhibitor (SP600125) recovered the ciliated cells and cilium length in PM2.5-treated cells. Moreover, c-Jun inhibitor suppressed upregulation of SPRR3 in PM2.5-treated cells. Taken together, our finding suggested that PM2.5 inhibits ciliogenesis by increasing SPRR3 expression via c-Jun activation in RPE cells and keratinocytes.

## Introduction

Atmospheric pollutants cause serious health problems. The premature death of 3.7 million people annually worldwide is linked to air pollution^1^. Particulate air pollutants include asian dust storm particles (ADSPs) and fine particulate matters (PMs), with the latter comprising coarse and fine fractions with aerodynamic diameters <10 and 2.5 μm (PM10 and PM2.5, respectively)^2^. PMs are heterogeneous pollutants composed of several molecules, including toxic heavy metals, ionic elements, and polycyclic aromatic hydrocarbons, which constitute the primary hazardous components of air pollutants. Recently, numerous deaths and other health problems have been reported to be associated with particulate pollution^3^.

PMs are known to cause epithelium injury and endothelial dysfunction^4,5^. Since PMs penetrate the nasal cavity and bronchial cilia, PMs cause inflammation, asthma, and chronic bronchitis^4,5^. In addition, the airborne particles could cause the development and aggravation of symptoms of skin diseases such as atopic dermatitis and psoriasis by increasing oxidative stress and inflammatory response^6,7^. Moreover, PMs also induce eye injury and increase the risk of cardiovascular damage and neurotoxicity^8–12^. The skin consists of two layers, the epidermis and dermis, which are involved in protection, regulation, and sensation. The main function of skin is to act as a physical barrier to protect the interior from harmful the effects of ultraviolet (UV) radiation, microorganism, and toxic molecules. Skin is intensively connected with nerve system and senses environmental changes^13^.

Because the primary cilium is a major cellular sensory organelle that functions as an antenna for sensing extracellular information, they mediate the interactions between cells and external stimuli including chemical and mechanical signals^14,15^. Primary cilia are highly conserved, dynamic, microtubule-based organelles, which emanate from the surface of many human cell types. The major role of primary cilia is to recognize extracellular signals such as growth factors, nutrients, and hormones^16,17^. The process of formation of primary cilia, called ciliogenesis, is regulated by the intraflagellar transport (IFT) protein complexes, IFT-A and IFT-B^18^. The cilium membrane harbors a number of receptors, ion channels, and signaling components such as sonic hedgehog (Shh) and Wnt receptors^14^; thus, primary cilia play an important role in signal transduction during development, cell migration, the cell cycle, and apoptosis^19,20^. As ADP-ribosylation factor-like protein 13B (ARL13B), a small GTPase of the Arf/Arl family, and Smoothened (Smo) are specifically localized to primary cilia and regulate Shh^21,22^, these proteins are wildly used as a marker for primary cilia. Ciliogenesis is induced by serum starvation or highly confluent cell culture conditions^23^. Recent studies showed that ciliogenesis is promoted by various cellular stresses, including UV radiation, heat shock, actin destabilization, and loss of mechanical stresses as well as serum starvation^24,25^. Therefore, ciliogenesis is highly linked with the arrest of cell growth and proliferation. Furthermore, differentiation of stem cells requires the presence of primary cilia and associated IFT^26^. It was recently shown that the absence of primary cilia inhibits differentiation of mesenchymal stem cells^26,27^.

Since primary cilia play important roles in tissue development, cell differentiation, and homeostasis, defects in their formation are associated with a wide range of human disorders, including various ciliopathies^19^. In this study, we hypothesized that PM as a toxic molecule may induce dysfunction via primary cilia in the skin, and found that ciliogenesis was increased in differentiated normal human epidermal keratinocytes (NHEKs) and PM2.5 negatively regulated ciliogenesis. In addition, PM2.5 increased the expression of small proline rich protein 3 (SPRR3) via activation of c-Jun in retinal pigment epithelium (RPE) cells and keratinocytes. Our results provide insight into the molecular and cellular bases for tissue damage caused by exposure to atmospheric pollutants.

## Results

### PM2.5 negatively regulates ciliogenesis in NHEKs and RPE cells

**Figure 1:**
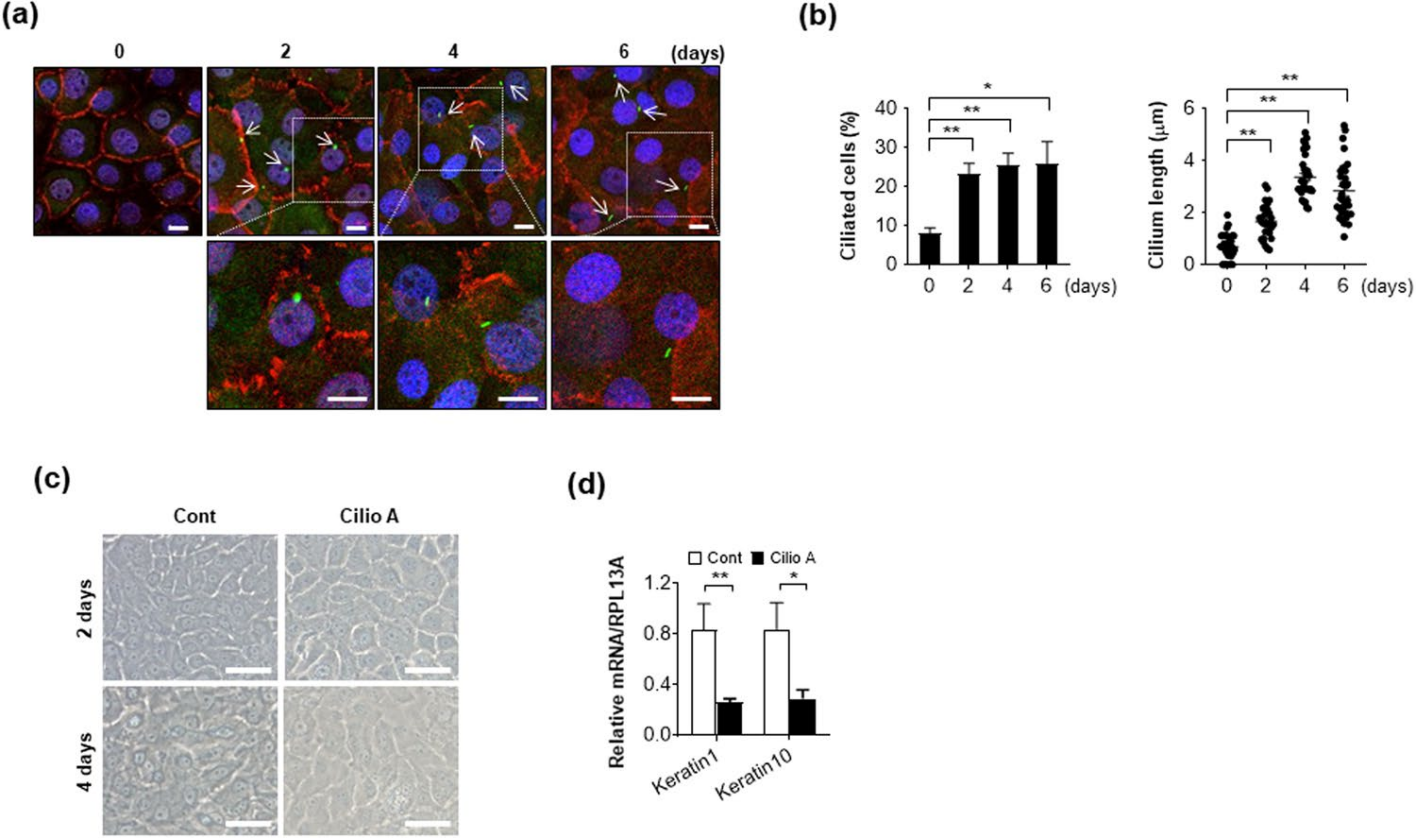
Differentiation induces ciliogenesis in normal human epidermal keratinocytes (NHEKs). (**a,b**) NHEKs grown to confluence were cultured for 6 days. Cells were stained with E-cadherin (red), ARL13B (green), and DAPI (blue) to obtain cell images (*lower*: enlarged image, scale bar = 10 μm) (**a**). Then, both ciliated cells and cilium length were determined as described in Material and Method part (**b**). (**c,d)** NHEKs grown to confluence were cultured for 4 days with or without ciliobrevin A1 (Cilio A, 5 uM). The cells were imaged with a bright microscopy (scale bar = 50 μm) (**c**), and harvested to analyze the level of Keratin1 and Keratin10 by using RT-PCR assay (**d**). Data were obtained from at least three independent experiments and values are means ± SE (*p < 0.05 and **p < 0.01).

Primary cilia have various functions and play a role in cell differentiation, which can be initiated by cell-cell contact. To investigate the presence of primary cilia on differentiated keratinocytes, NHEKs were cultured under confluent condition for 2, 4, and 6 days to induce cell differentiation. Primary cilia formation by NHEKs was observed by immunostaining with ARL13B, which is a small G protein localized in the primary cilia. Both the number of cells with primary cilia and length of the cilium were significantly increased in differentiation-induced keratinocytes (Fig. 1a,b). Then, we further investigate the effect of primary cilia on differentiation in NHEKs by treatment with ciliobrevin A1/Hedgehog pathway inhibitor 4 (HPI4). Ciliobrevin A1 is the first specific small-molecule antagonist of the cytoplasmic dyneins, and was reported to chemically inhibit primary cilium formation^28^. During cell differentiation, the expression of various differentiation markers, such as involucrin, loricrin, keratin1, and keratin10 increase with time^29^. We found that differentiation markers including keratin1 and keratin10 was notably decreased in ciliobrevin A1-treated keratinocytes (Fig. 1c,d).

**Figure 2:**
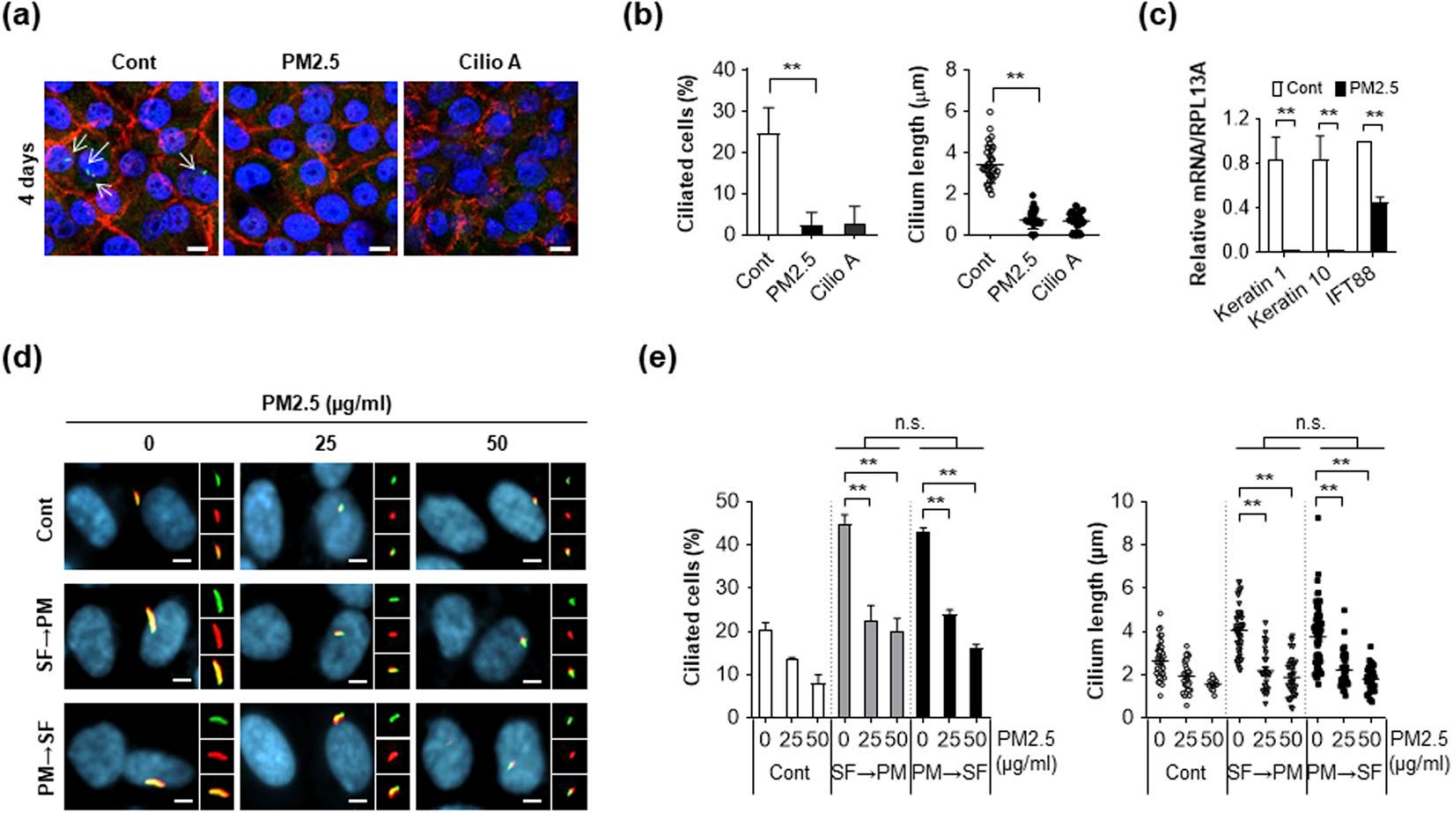
PM2.5 suppresses ciliogenesis and in NHEKs and RPE cells. (**a–c**) NHEKs grown to confluence were cultured for 4 days with either PM2.5 (50 µg/mL) or ciliobrevin A1 (5 μM). Cells were stained with E-cadherin antibody (red), ARL13B antibody (green), and DAPI (blue) to acquire cell images (**a**). Both ciliated cells and cilium length were determined using fluorescence microscopy (**b**). Differentiation of NHEKs was examined using RT-PCR asssay with indicated markers (**c**). (**d,e**) RPE cells pretreated with PM2.5 (50 µg/mL) were further incubated in serum-free medium (SF) condition for 12 hours [PM (24 h) → SF (12 h)]. Or RPE cells cultured in SF media for 12 h were further exposed to PM2.5 [(25 and 50 µg/mL, SF (12 h) → PM (24 h)]. Then the cells were stained with acetylated tubulin antibody (AT, red) and ARL13B antibody (green). (yellow: red-green merged image) (**d**). (**e**) Both ciliated cells and cilium length were counted using fluorescence microscopy. Data are means ± standard error (SE) of three independent experiments (**p < 0.01, scale bar = 5 μm).

Keratinocytes form the outermost layer of the skin and provide a barrier against environmental damage. External environmental stimuli, including chemical, mechanical, and paracrine signals can regulate ciliogenesis and, therefore, we examined the effect of PM2.5 on cilium formation in keratinocytes. NHEKs grown to confluence were cultured for 4 days in the presence or absence of PM2.5. Interestingly, the number of cells with primary cilia and their length were notably decreased in PM2.5-treated NHEKs when compared with the untreated control cells (Fig. 2a,b). Treatment with ciliobrevin A1 suppressed cell differentiation as well as ciliogenesis in keratinocytes (Fig. 2a,b). Consistently, we found that the mRNA expression levels of keratinocyte differentiation markers and primary cilia marker, IFT88 were also reduced by PM2.5-treated NHEKs (Fig. 2c). Taken together, these results suggested that PM2.5 regulates ciliogenesis in differentiated keratinocytes.

Then, we further confirmed the effect of PM2.5 on ciliogenesis in other cell types. Because RPE cells show clear primary cilium, the cells are widely used to study the role of primary cilia^23^. To confirm the effect of PM2.5 on ciliogenesis, RPE cells were cultured in serum-free conditional medium either before or after PM2.5 treatment. To image the primary cilia, the cells were stained with both anti-acetylated tubulin antibody and ARL13B antibody. Consistent with HNEKs, incubation of RPE cells in serum-free condition highly increased the cilium length and cell numbers (Fig. 2d,e). However, increased ciliogenesis by serum deprivation was significantly blocked by PM2.5 in RPE cells, suggesting that PM2.5 accelerated disassembly of primary cilia (Fig. 2d,e). Moreover, we found that pre-treatment with PM2.5 efficiently reduced ciliogenesis in serum-deprived RPE cells (Fig. 2d,e), further indicating that PM2.5 decreased ciliogenesis and increased the degradation of primary cilia. Taken together, these results suggest that PM2.5 negatively regulates primary cilia in RPE cells and NHEKs.

### PM2.5 upregulates SPRR3 expression and ectopic expression of SPRR3 blocks ciliogenesis

**Figure 3:**
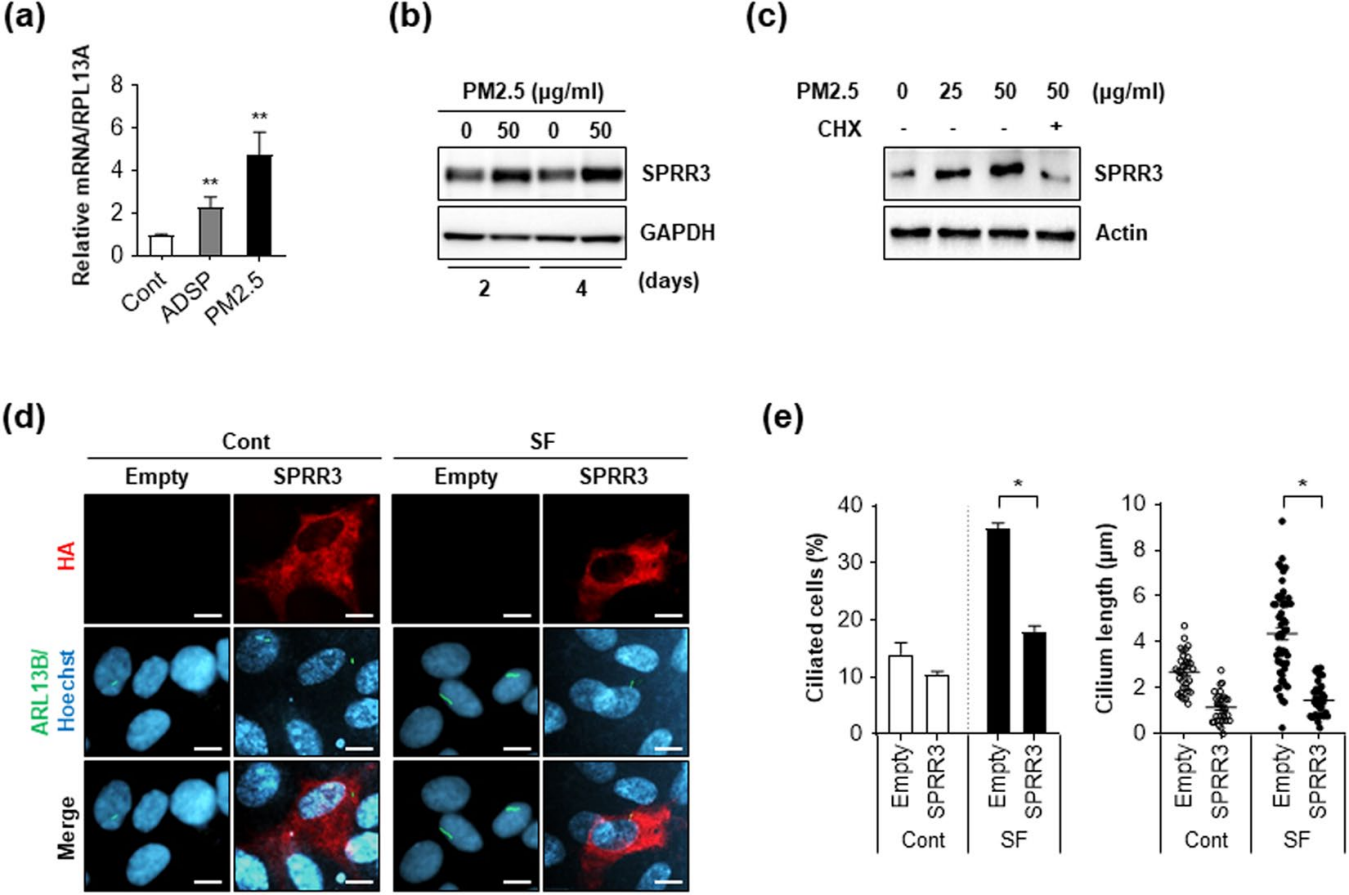
PM2.5 upregulates SPRR3 to suppress ciliogenesis. (**a**) NHEKs were exposed to either ADSP (25 ug/mL) or PM2.5 (50 µg/mL) for 48 h. Then, mRNA level of SPRR3 was determined using a RT-PCR analysis. (**b**) NHEKs were exposed to PM2.5 for either 2 days or 4 days, then cells were analyzed by Western blotting with SPRR3 antibody. GAPDH was used as an internal loading control. (**c**) RPE cells were exposed to PM2.5 for 24 h in the presence or absence of cycloheximide (CHX, 10 µg/mL) for 14 h and were analyzed using Western blotting with SPRR3 and Actin antibodies. (**d,e**) Retinal pigment epithelium (RPE) cells transiently transfected with pHA-SPRR3 were incubated with normal culture medium (Cont) or serum-free condition (SF). (**d**) After 48 h, cells were imaged by staining with anti-HA antibody (red), ADP-ribosylation factor-like protein 13B (ARL13B) antibody (green), and Hoechst dye (blue). (**e**) Then, both ciliated cells and cilium length were counted under a fluorescence microscopy. Data are means ± standard error (SE) of three experiments (*p < 0.05).

Our previous transcriptome analysis in keratinocytes showed that treatment with PM2.5 upregulated several SPRR family proteins, such as SPRR2, SPRR3, and SPRR4^30^. In addition, SPRR3 was screened as a potential ciliogenesis regulator from a small-scale short interfering RNA (siRNA) library screening, thus, we further investigated the effect of SPRR3 on ciliogenesis in PM2.5-treated cells. To confirm the mRNA-sequencing (RNA-seq) analysis, we examined the expression of SPRR3 in PM2.5-treated keratinocytes. Consistent with our previous data, both mRNA and protein levels of SPRR3 were remarkably increased in PM2.5-treated NHEKs (Fig. 3a,b). ADSP also upregulated SPRR3 expression in NHEKs (Fig. 3a). Furthermore, treatment with a protein translation inhibitor, cycloheximide, blocked SPRR3 expression in PM2.5-treated RPE cells (Fig. 3c), suggesting that PM2.5 upregulated SPRR3 expression in keratinocytes and RPE cells. Moreover, we observed that ectopic expression of SPRR3 in RPE cells decreases the serum deprivation-induced ciliogenesis (Fig. 3d,e). Taken together, these results suggest that the upregulation of SPRR3 induced by PM2.5 can contribute to suppression of ciliogenesis.

### Depletion of SPRR3 enhances ciliogenesis in PM2.5-treated cells

**Figure 4:**
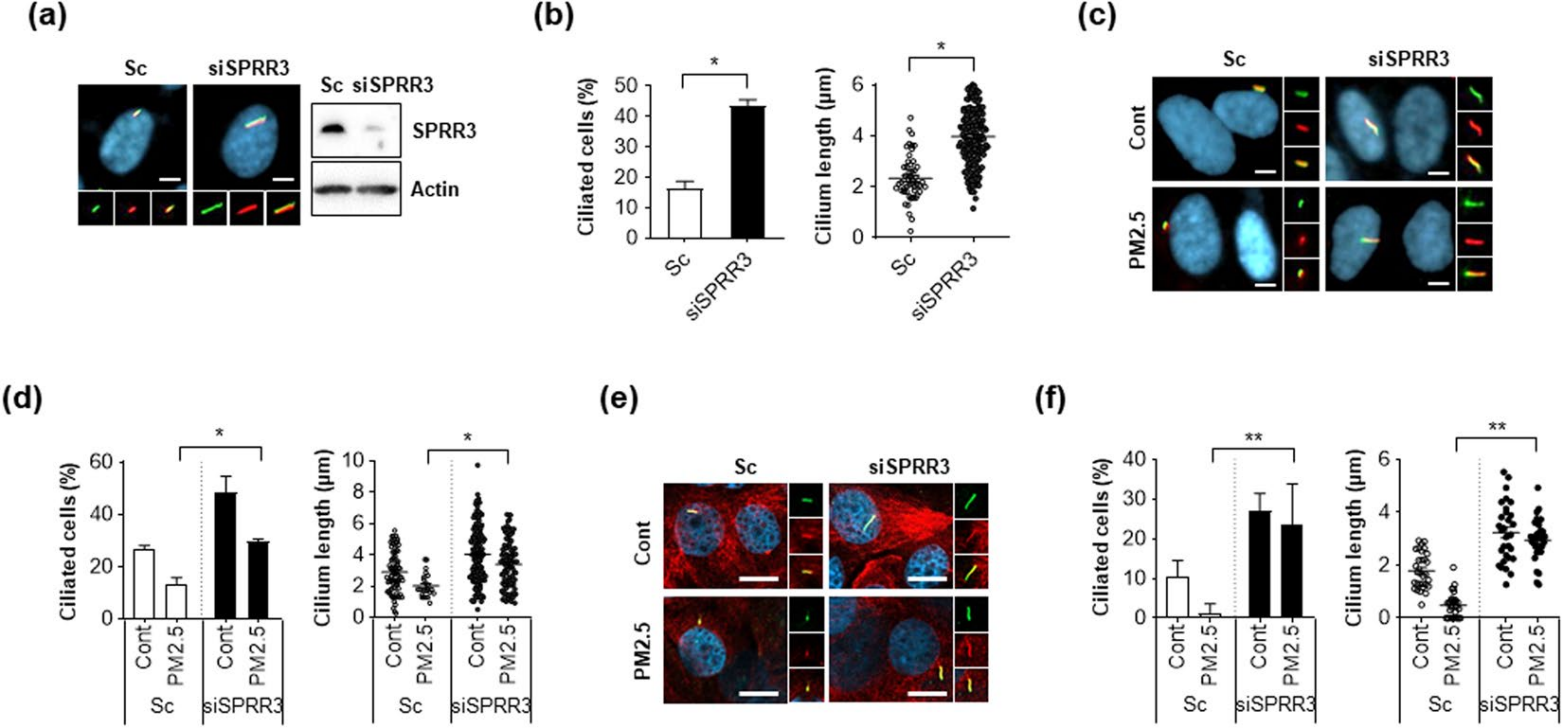
Depletion of SPRR3 restores reduced primary cilia in PM2.5-treated cells. (**a,b**) Retinal pigment epithelium (RPE) cells were transiently transfected with small interfering RNA (siRNA) targeting SPRR3 (siSPRR3) or scrambled siRNA (Sc). After 3 days, cells were stained with ARL13B antibody (green), acetylated tubulin (red) and Hoechst dye (blue) (yellow: red-green merged image) (scale bar = 5 μm) (**a**). Then both ciliated cells and cilium length were counted using fluorescence microscopy (**b**). (**c,d**) RPE cells transiently transfected with siSPRR3 or Sc were treated with PM2.5 (50 µg/mL) for 24 h, were stained with acetylated tubulin antibody (red), ARL13B antibody (green), and Hoechst dye (blue) (yellow: red-green merged image) (**c**, scale bar = 10 μm). (**d**) Then both ciliated cells and cilium length were counted using fluorescence microscopy. (**e,f**) NHEKs transiently transfected with siRPRR3 or Sc were treated with PM2.5 (50 µg/mL) for 24 h, and stained with acetylated tubulin (red), ARL13B (green), and DAPI (blue, scale bar = 10 μm). (**f**) Then, both ciliated cells and cilium length were counted using fluorescence microscopy. Data are from at least three independent experiments and are means ± standard error (SE, *p < 0.05 and **p < 0.01).

To further investigate the function of SPRR3 in ciliogenesis, we performed a loss-of-function experiment in which SPRR3 expression was silenced using siRNA. Depletion of *SPRR3* enhanced both ciliated cells and their length in RPE cells (Fig. 4a,b). Additionally, knockdown of *SPRR3* efficiently reversed decreased primary cilia induced by PM2.5 in RPE cells (Fig. 4c,d). In addition to RPE cells, depletion of *SPRR3* expression also restored loss of primary cilia in NHEKs (Fig. 4e,f), implying that SPRR3 was closely involved in the regulation of ciliogenesis in PM2.5-treated cells.

### Activation of c-Jun by PM2.5 increases SPRR3 expression, but inhibition of c-Jun recovers ciliogenesis in PM2.5-treated cells

**Figure 5:**
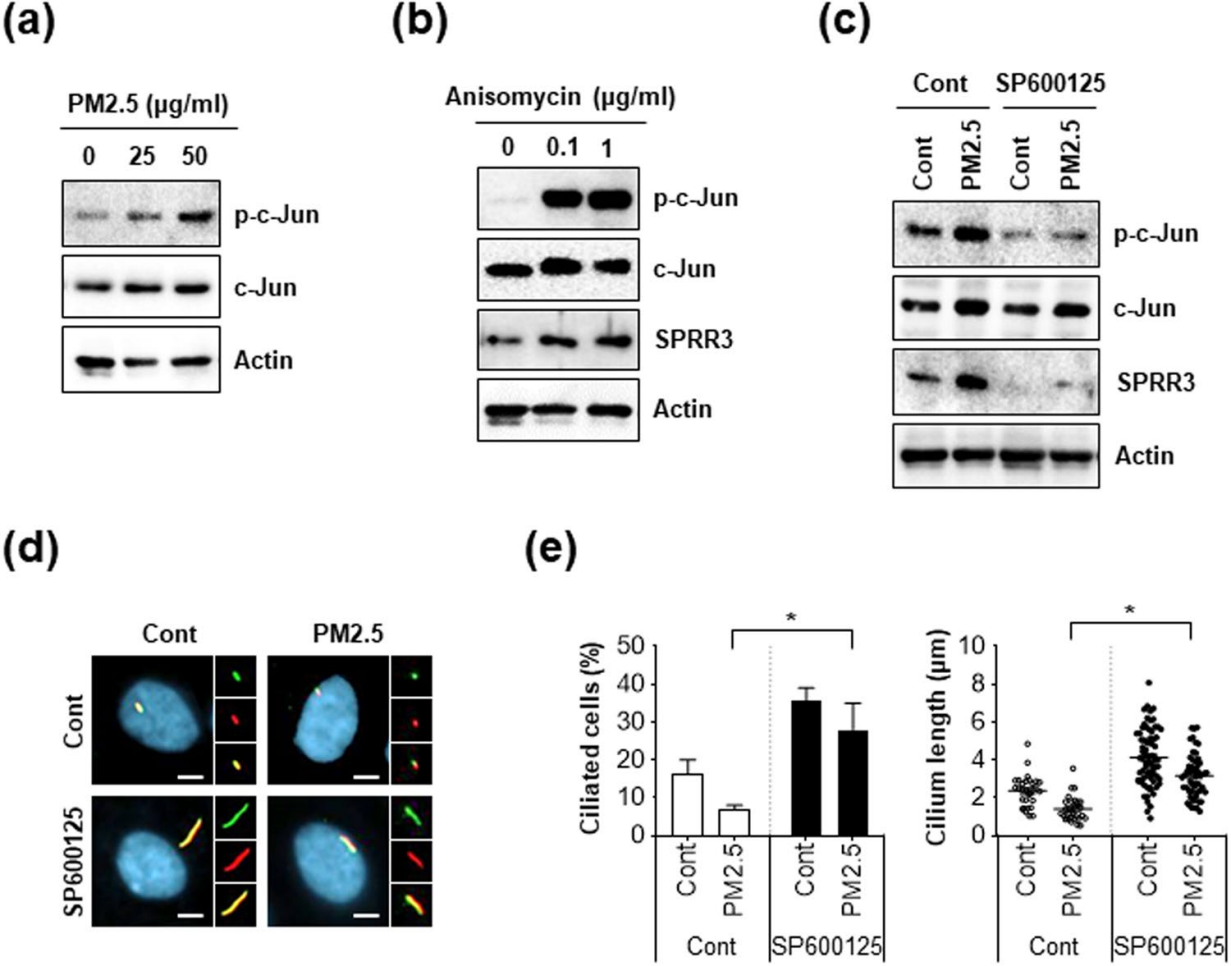
Inhibition of C-Jun induces ciliogenesis by decreasing SPRR3 expression in PM2.5-treated RPE cells. (**a,b**) RPE cells were incubated PM2.5 (25 and 50 µg/mL) for 24 h or anisomycin (0.1 and 1 µg/mL) for 1 h and expression of indicated proteins was detected using western blot analysis. (**c,d**) RPE cells were treated with PM2.5 (50 µg/mL) in the presence or absence of SP600125 (10 µM) for 24 h. (**c**) Cells were stained with acetylated tubulin antibody (red) and ARL13B antibody (green) Hoechst dye (blue, yellow: merged image, scale bar = 5 μm). (**d**) Then, both ciliated cells and cilium length were determined using fluorescence microscopy. (**e**) RPE cells were exposed to PM2.5 (50 µg/mL) in the presence or absence of SP600125 (10 µM) for 24 h. Then, cells were analyzed using western blotting with indicated antibodies. Data are means ± standard error (SE) of at least three independent experiments (*p < 0.05).

**Figure 6:**
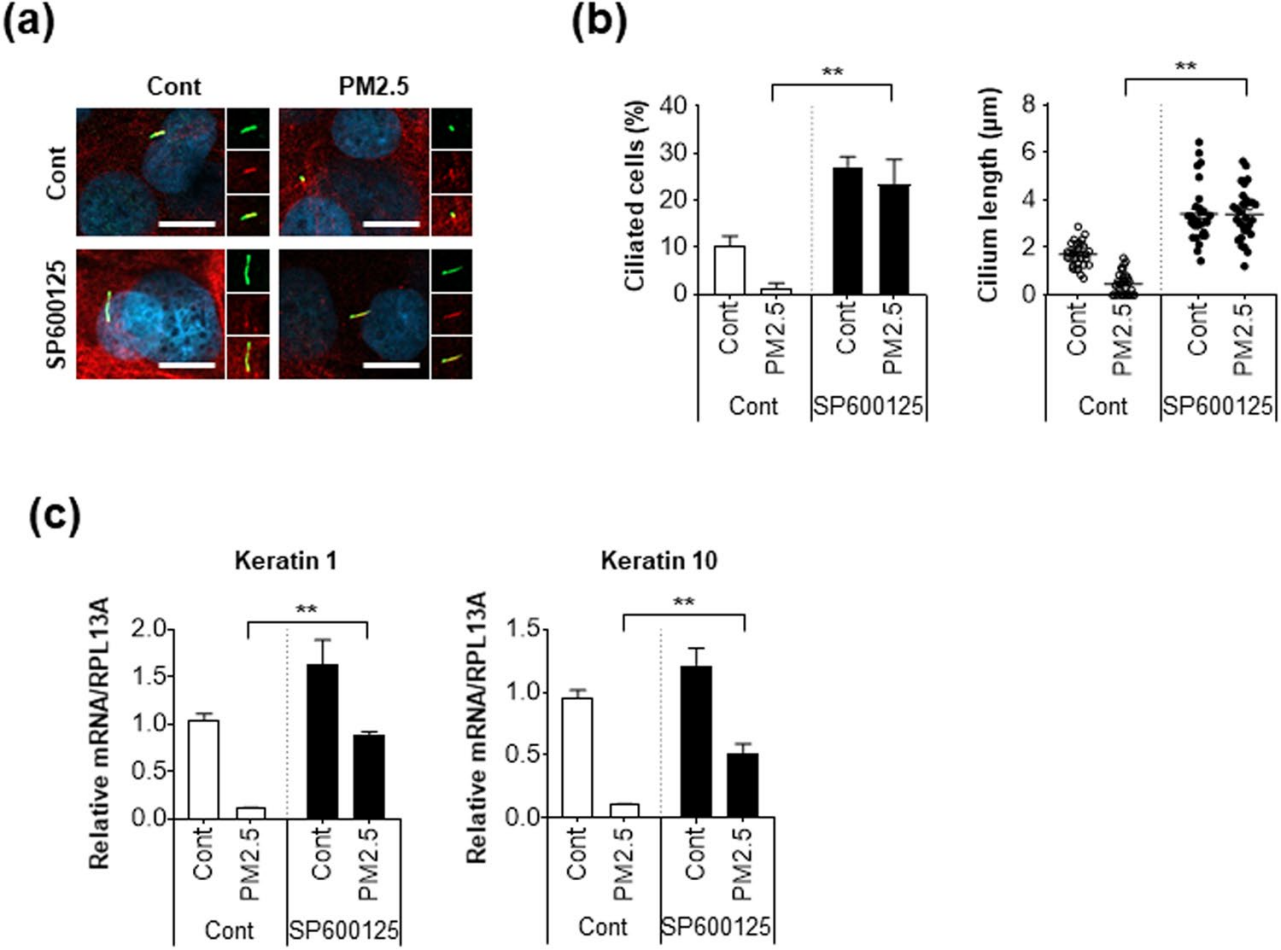
PM2.5-mediated down-regulation of ciliogenesis and differentiation was restored by inhibition of c-Jun in keratinocytes. (**a**–**c**) NHEKs were treated with PM2.5 (25 µg/mL) with or without SP600125 (10 µM) for 24 h (**a**). Then, cells were stained with acetylated tubulin antibody (red), ARL13B (green), and DAPI (blue, scale bar = 10 μm). Then, both ciliated cells and cilium length were counted under a fluorescence microscopy (**b**), and harvested to analyze the level of Keratin1 and −10 by using RT-PCR (**c**). Data are means ± standard error (SE) of at least three independent experiments (*p < 0.05 and **p < 0.01).

We further explored the potential molecular mechanism of PM2.5-regulated ciliogenesis. Interestingly, it was recently reported that SPRR3 expression is upregulated by the transcriptional factor, c-Jun, which is activated by phosphorylation^31^. In addition, we found that SPRR3 is increased in PM2.5-treated cells. Therefore, we further examined c-Jun activation in PM2.5-treated cells and found that phosphorylation of c-Jun was notably increased by PM2.5 treatment (Fig. 5a). In addition, treatment with anisomycin, a potent activator of c-Jun, highly induced SPRR3 expression as well as c-Jun phosphorylation (Fig. 5b). In contrast, treatment with a c-Jun inhibitor (SP600125) strongly blocked the increased expression of SPRR3 in PM2.5-treated cells (Fig. 5c). More importantly, immunostaining assay with primary cilia revealed that down-regulated ciliogenesis in PM2.5-treated cells was further recovered by inhibition of c-Jun (Fig. 5d,e). We additionally confirmed the effect of c-Jun activation by PM2.5 in NHEKs. Consistent with RPE cells, inhibition of c-Jun by treatment with SP600125 suppressed the loss of primary cilia by PM2.5 exposure in NHEKs (Fig. 6a,b). Accordingly, SP600125 treatment significantly recovered down-regulated differentiation markers such as Keratin1 and Keratin10 in PM2.5-treated NHEKs (Fig. 6c). Taken together, these results suggest that c-Jun-mediated SPRR3 expression negatively regulated ciliogenesis and differentiation in PM2.5-treated keratinocytes.

## Discussion

Many toxicological and epidemiological studies have suggested that PMs negatively exert various human physiological systems, including respiratory system, circulatory system, nervous system, and integumentary system^32–34^. Among them, skin is directly exposed to PMs. Therefore, PM can easily penetrate the skin and induce abnormal skin states such as allergic reactions, aging, and delayed wound healing^35,36^. PMs can exert skin barrier dysfunction by decreasing filaggrin, which is a major structural protein in maintain the skin barrier function and influences allergic skin disease via Cox2 expression^37^. In addition, PMs also aggravate skin damage by increasing Ca^++^ influx which stimulates production of proinflammatory such as cytokines interleukin-1β and TNF-α in keratinocytes^38^.

Primary cilia play various roles in the skin such as mediating the initiation of hair follicle morphogenesis^39^. In addition, increased ciliogenesis, which activates the Smo-signalling pathway suppresses skin pigmentation in melanocytes^40^. Epidermal differentiation is suppressed by ciliary defective mutants and the elimination of cilia induces hyperproliferation of keratinocytes in mice during skin development^41^. In this study, we found that the formation of primary cilium is involved in differentiation in NHEKs (Fig. 1). Our findings suggest that primary cilia possibly are linked with cell differentiation in skin. The SPRR family members are known as cross-bridging proteins between other structural molecules just beneath the plasma membrane, which reinforce the cornified envelope of the skin epidermis. In this study, we found that the mRNA expression of SPRR family proteins was increased in PM2.5-treated cells, which decreased primary cilia formation. Among the SPRR family proteins, SPRR3 contributed to the alteration of primary cilia in keratinocytes in response to PM2.5 (Figs 2 and 3). Nonetheless, the upregulation of SPRR3 by PM has not been elucidated. Hu *et al*.^42^ recently reported that cigarette smoke transcriptionally increases SPRR3 expression in human bronchial epithelial (HBE) cells by favouring c-Jun/Fra1 heterodimerization^42^. In response to injury induced by chronic cigarette smoke exposure, HBE cells express various proteins associated with keratinized squamous cells^43^. Cigarette smoke is the main source of PM constituting indoor air pollution, which is a risk factor for chronic obstructive pulmonary diseases. Meaningfully, we also found that PM2.5 upregulated SPRR3 expression in an exposure-dependent manner in keratinocytes and RPE cells. In addition, inhibition of c-Jun activation blocked SPRR3 upregulation in PM2.5-treated cells (Fig. 5). Together with our findings, these results strongly indicated that the expression of SPRR3 is mainly regulated by c-Jun activation in PM-exposed cells.

The results of our study showed that loss of SPRR3 induces ciliogenesis, whereas SPRR3 overexpression had the opposite effect. Although the precise regulatory mechanism was not elucidated, we recently showed that ectopic expression of SPRR3 increased AKT phosphorylation in SW480 colorectal cancer cells^44^. The AKT pathway regulates various intracellular signaling events related to cell proliferation and differentiation. AKT is a component of the primary cilium maintenance signaling network. Hamamoto *et al*.^45^ reported that AKT phosphorylation is an important stage in melanin-concentrating hormone (MCH)-induced cilia length shortening in RPE cells^45,46^. Treatment with LY294002 and wortmannin, inhibitors of phosphoinositide 3-kinase (PI3K), which is an upstream AKT activator, significantly blocked MCH-induced cilia shortening. In addition, glycogen synthase kinase 3 β (GSK3β) and mammalian target of rapamycin (mTOR) are downstream of AKT, and were reported to be regulators of ciliogenesis^47,48^. However, the role of the AKT pathway in ciliogenesis is not simple and seems to be dependent on the ciliated cell types. For instance, inactive phosphorylation of GSK3 β by ATK was reported to occur in leptin-induced ciliogenesis in N1 hypothalamic neurons^49^. Depletion of phosphatase and tensin homolog (PTEN) and GSK3β increased ciliogenesis in leptin-treated cells. Therefore, further studies are needed to clarify the contribution of AKT signaling to the regulation of primary cilia by SPRR3.

In conclusion, we found that PM2.5 disrupts the formation of primary cilia via c-Jun-mediated regulation of SPRR3 expression in ciliated cell types of the skin and eye. These findings provide molecular-level insight into the damaging effects of PM on tissues exposed to the atmosphere as well as a justification for stricter regulations to limit human exposure to harmful air pollutants.

## Materials and Methods

### Cell culture

Normal human epidermal keratinocytes (NHEKs) from the neonatal foreskin donors were purchased from Lonza (Basel, Switzerland) and were cultured in KBM-GOLD medium supplemented with insulin, human epidermal growth factor, bovine pituitary extract, hydrocortisone, epinephrine, transferrin, and gentamicin/amphotericin B (all supplements from Lonza). The cells were serially passaged at 70–80% confluence, and experiments were performed using subconfluent cells within two or three passages. NHEKs grown to confluence were cultured with PM 2.5 (50 μg/mL) in KBM-Gold medium without supplements for 2 or 4 days. Human telomerase-immortalized retinal pigmented epithelial (RPE) cells were provided by Dr. Joon Kim (KAIST, Korea)^25^. RPE cells were cultured in Dulbecco’s Modified Eagle’s medium supplemented with 10% fetal bovine serum and 1% penicillin-streptomycin (Invitrogen, Carlsbad, CA, USA).

### Reagents and siRNA transfection

PM2.5 was collected on a Teflon filter (Zefluor, Pall Life Science, Ann Arbor, MI, USA) using a low-volume air sampler consisting of a cyclone (2.5-μm size cut, URG-2000-30EH), two upstream denuders (annular, URG-2000-30x242-3CSS), Teflon filter (Zefluor^TM^ 2.0 μm Pall Life Sciences, Mexico), backup filter, and backup denuder in the series^50^. The collection started at approximately 10:00 a.m., the filters were replaced every 24 h, and the flow rate was 16.7 L/min. The sampling region was located 35 km southeast of downtown Seoul, Korea (37.34°N, 127.27°E, 167 m above sea level). Detailed information of the location and collection methods is provided in our previous report^43^. The filter was sonicated in ethanol (EtOH) for 30 min, the EtOH was evaporated, and then the PM2.5 was resuspended in deionized water. ADSPs were also sampled from the roof of the Korea University Science Building, located in the north-eastern region of Seoul, Korea (37.35°N, 127.01°E). The PMs were collected using a high-capacity air collector with a quartz filter, as described previously^51^.

All chemical reagents, including ciliobrevin A1 (2,4-Dichloro-a-(3,4-dihydro-4-oxo-2(1H)-quinazolinylidene)-β-oxo-benzenepropanenitrile/Hedgehog pathway inhibitor 4), Hoechst 33342, 4′,6-diamidino-2-phenylindole (DAPI), cycloheximide, anisomycin, and SP600125 were purchased from Sigma-Aldrich (St. Louis, MO, USA). Previously validated siRNAs targeting SPRR3 (5ʹ-CAGACAAGCCCUUGAGAA-3ʹ)^52^ was synthesized by Genolution (Seoul, Korea). Cultured cells were transfected with siRNA using Lipofectamine^®^ RNAi MAX (Invitrogen, Carlsbad, CA, USA) according to the manufacturer’s instructions. After incubation with siRNA for 48 h, the cells were treated with each chemical for 24 h.

### RNA extraction and quantitative real-time reverse transcription polymerase chain reaction (q-RT-PCR)

Total RNA was isolated using TRIzol reagent (Invitrogen) according to the manufacturer’s instructions. The RNA concentration was spectrophotometrically determined, and RNA integrity was analyzed using a BioAnalyzer 2100 (Agilent Technologies, Santa Clara, CA, USA); 4 μg were reverse transcribed into cDNA using SuperScript III reverse transcriptase (Invitrogen). The expression levels of target genes were evaluated by qRT-PCR using TaqMan technology (7500 Fast; Applied Biosystems, Foster City, CA, USA). The cycling protocol was as follows: 95 °C for 10 min, followed by 50 cycles of 95 °C for 15 s and 60 °C for 1 min. The target genes were as follows: keratin 1, Hs00196158_m1; keratin 10, Hs00166289_m1; IFT88, Hs00544051_m1; and SPRR3, Hs00271304_m1. Ribosomal protein L13A (Hs04194366_g1; Applied Biosystems) was used to normalize the expression levels of target genes.

### Western blot analysis

All lysates were prepared in 2 × Laemmli sample buffer (62.5 mM Tris-HCl, pH 6.8, 25% [v/v] glycerol, 2% [w/v] sodium dodecyl sulphate [SDS], 5% [v/v] β-mercaptoethanol, and 0.01% [w/v] bromophenol blue, Bio-Rad, Hercules, CA, USA). All cellular proteins were quantified using the Bradford solution (Bio-Rad) according to the manufacturer’s instructions. The samples were then separated using SDS-polyacrylamide gel electrophoresis (PAGE) and transferred onto a polyvinylidene fluoride (PVDF) membrane (Bio-Rad). After blocking with 4% (w/v) skim milk in Tris-buffered saline plus Tween (TBST; 25 mM Tris, 140 mM sodium chloride [NaCl], and 0.05% [v/v] Tween^®^ 20), the membranes were incubated overnight with the following specific primary antibodies: HA (1:3000, Santa Cruz), SPRR3 (1:3000, Proteintech), Actin (1:10000, EMD Millipore), GAPDH (1:10000, Cell Signaling Technology), phosphor-c-Jun (1:1000 Cell Signaling Technology) and c-Jun (1:3000 Cell Signaling Technology). For protein detection, the membranes were incubated with horseradish peroxidase (HRP)-conjugated secondary antibodies and the signals were detected using SuperSignal^®^ West Dura HRP detection kit (Pierce, Rockford, IL, USA).

### Immunofluorescence analysis using confocal microscopy

Cells cultured on Lab-Tek™ glass chamber slides (Thermo Scientific, Waltham, MA, USA) were washed with cold phosphate-buffered saline (PBS), fixed for 20 min with 4% (w/v) paraformaldehyde, washed twice with PBS, and incubated in PBS containing 0.1% (v/v) Triton™ X-100 (PBS-T) for 3 min. The cells were further washed three times with PBS-T and then incubated with the following antibodies: anti-acetylated-tubulin (1:1000, Sigma-Aldrich), anti-cadherin (1:1000, Santa Cruz Biotechnology, Santa Cruz, CA, USA), anti-HA (1:1000, Santa Cruz Biotechnology), and anti-ARL13B (1:1000, Proteintech) diluted in PBS-T containing 5% (v/v) normal goat serum for 1 h at room temperature. After washing with PBS, the cells were incubated with appropriate conjugated secondary antibodies and then mounted on glass slides to observe the fluorescence using a confocal laser scanning microscope (model LSM510; Carl Zeiss Microimaging Inc., Thornwood, NY, USA).

### Measurement of increased cilium number and length

Changes in the cilium numbers in cells were measured by counting the immunofluorescent stained primary cilia. The cilium length was determined using the cell-Sense standard software (Olympus, Japan). The average cilium length was calculated using the Free-hand Line selection tool. The length of an individual cilium was examined from randomly selected cells, and the images were captured and digitized using the cell-Sense standard software and GraphPad Prism 8 (30–200 cells per experiment, n = 3).

### Statistical analysis

Data were obtained from at least three independent experiments and are presented as means ± standard error (SE). The results were statistically evaluated using one-way or two way analysis of variance using the statistical package for the social sciences (SPSS, software, version 21).

## Supplementary information

Western blot souce data
