# Supplementary material for: Fine particulate matter (PM2.5) inhibits ciliogenesis by increasing SPRR3 expression via c-Jun activation in RPE cells and skin keratinocytes
Source: Sci Rep. 2019 Mar 8;9:3994. doi: 10.1038/s41598-019-40670-y (PMC6408442; doi:10.1038/s41598-019-40670-y)

# Fine particulate matter (PM2.5) inhibits ciliogenesis by increasing SPRR3 expression via c-Jun activation in RPE cells and skin keratinocytes

Ji-Eun Bae[1,2†], Hyunjung Choi[3†], Dong Woon Shin[2], Hye-Won Na[3], Na Yeon Park[1], Joon Bum Kim[1], Doo Sin Jo[1], Min Ji Cho[4], Jung Ho Lyu[4], Jeong Ho Chang[5], Eunjoo H. Lee[2], Tae Ryong Lee[3], Hyoung-June Kim[3*], and Dong-Hyung Cho[1*]

Figure 3B

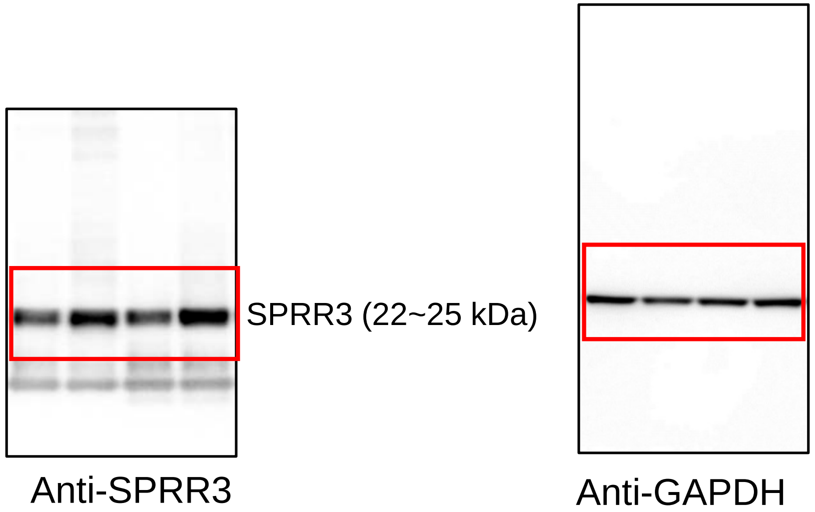


Figure 3C

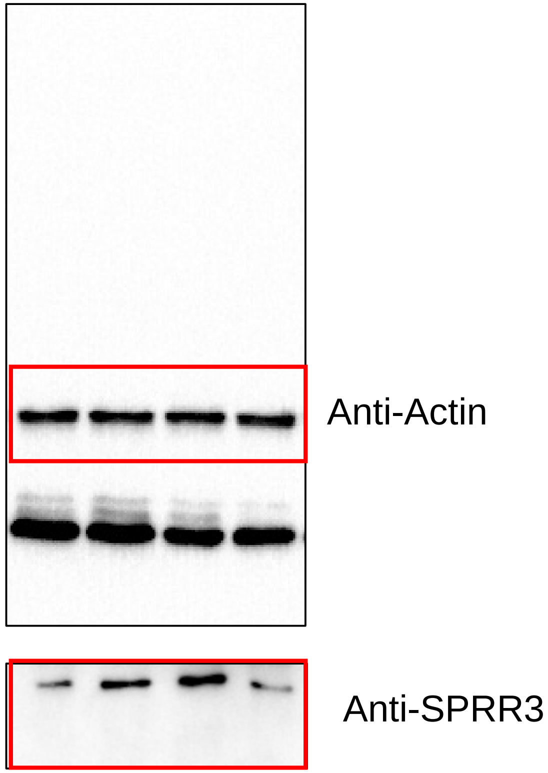

Figure 4A

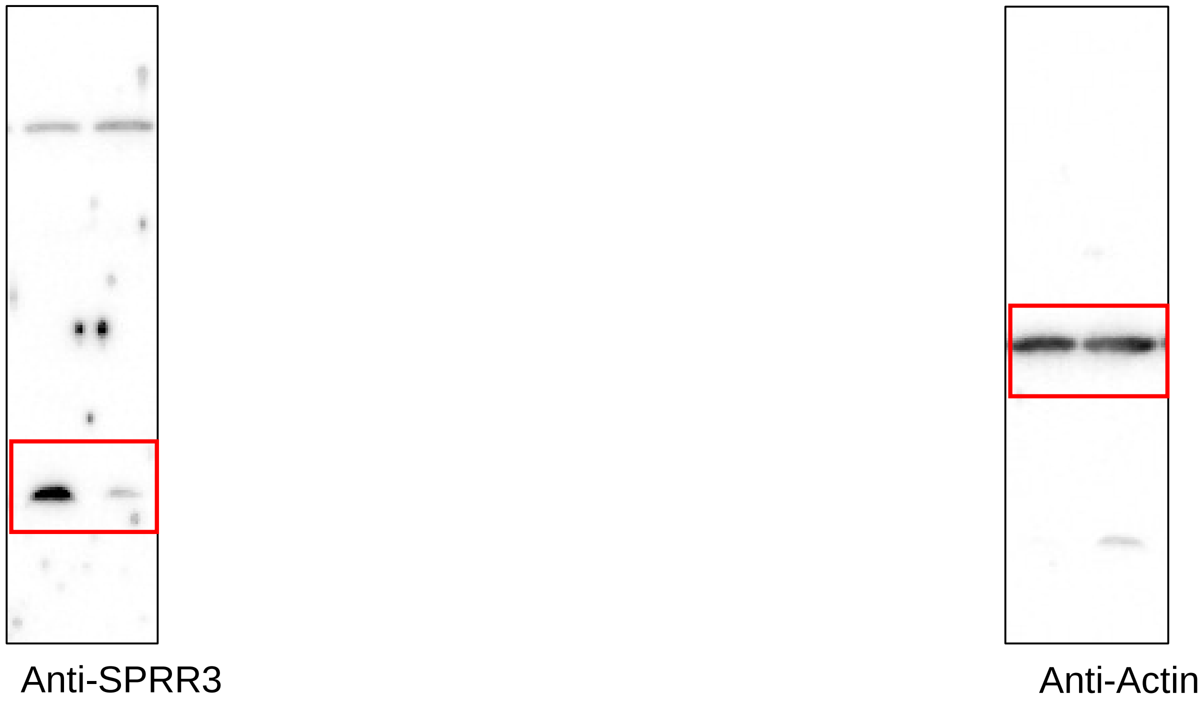

Figure 5A

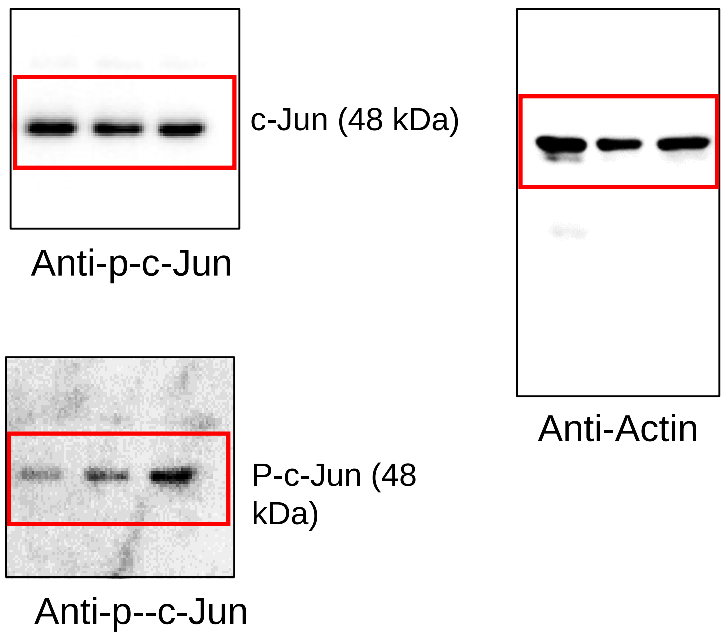


Figure 5B

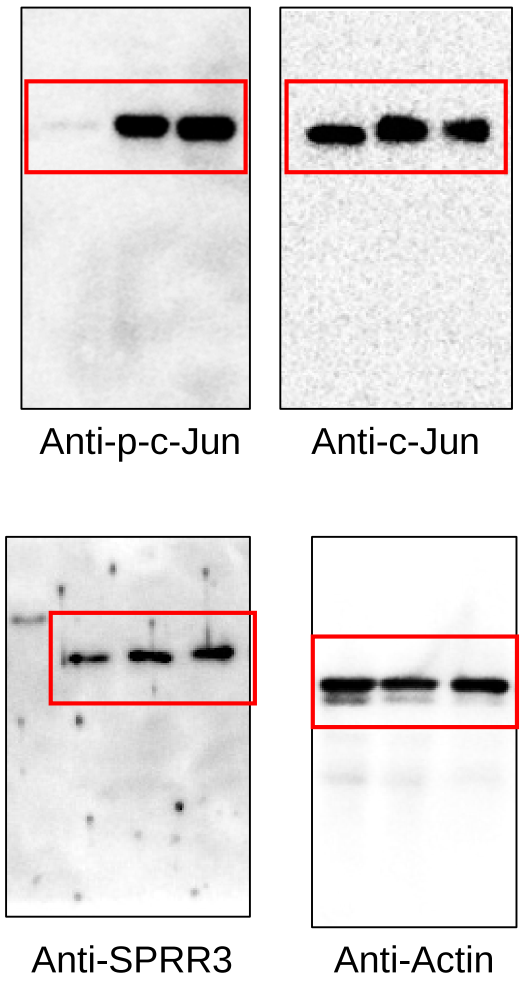


Figure 5C

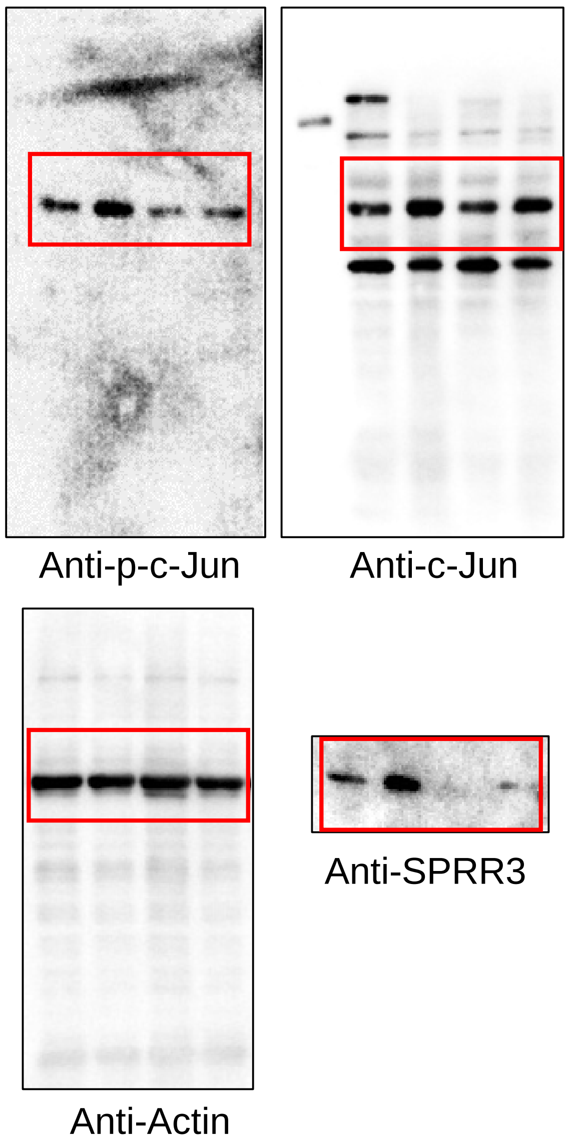

Supplement: Supplementary file 1 — Western blot souce data [file 41598_2019_40670_MOESM1_ESM.pdf]
